# Preparation of Calcined Zirconia-Carbon Composite from Metal Organic Frameworks and Its Application to Adsorption of Crystal Violet and Salicylic Acid

**DOI:** 10.3390/ma9040261

**Published:** 2016-03-31

**Authors:** Zubair Hasan, Dong-Wan Cho, In-Hyun Nam, Chul-Min Chon, Hocheol Song

**Affiliations:** 1 Department of Environment and Energy, Sejong University, Seoul 143-747, Korea; zubair.hasan.du@gmail.com (Z.H.); heavens83@hanmail.net (D.-W.C.); 2 Geologic Environment Division, Korea Institute of Geoscience and Minieral Resources, Daejeon 305-350, Korea; nih@kigam.re.kr (I.-H.N.); femini@kigam.re.kr (C.-M.C.)

**Keywords:** metal-organic frameworks, composites, adsorption, crystal violet, salicylic acid

## Abstract

Zirconia-carbon (ZC) composites were prepared via calcination of Zr-based metal organic frameworks, UiO-66 and amino-functionalized UiO-66, under N_2_ atmosphere. The prepared composites were characterized using a series of instrumental analyses. The surface area of the ZC composites increased with the increase of calcination temperature, with the formation of a graphite oxide phase observed at 900 °C. The composites were used for adsorptive removal of a dye (crystal violet, CV) and a pharmaceutical and personal care product (salicylic acid, SA). The increase of the calcination temperature resulted in enhanced adsorption capability of the composites toward CV. The composite calcined at 900 °C exhibited a maximum uptake of 243 mg·g^−1^, which was much greater than that by a commercial activated carbon. The composite was also effective in SA adsorption (102 mg·g^−1^), and N-functionalization of the composite further enhanced its adsorption capability (109 mg·g^−1^). CV adsorption was weakly influenced by solution pH, but was more dependent on the surface area and pore volume of the ZC composite. Meanwhile, SA adsorption showed strong pH dependence, which implies an active role of electrostatic interactions in the adsorption process. Base-base repulsion and hydrogen bonding are also suggested to influence the adsorption of CV and SA, especially for the N-functionalized composite.

## 1. Introduction

Recently, metal oxide-carbon composites have been gaining much attention because of their superior performances demonstrated in various environmental applications [1,2]. The textural properties of non-porous (or low surface area) metal oxides may undergo significant changes upon dispersion on porous carbons, which ultimately improves the material’s capability for a desired purpose. Among many inorganic oxides, zirconia (ZrO_2_) has attracted a great deal of attention for its wide application in different fields, such as adsorption [3,4], catalysis [5], and photo-degradation [6]. However, its low surface area limits the potential applicability of ZrO_2_, especially in catalysis and adsorption. Several approaches have been suggested to address this limitation, including incorporation of ZrO_2_ in porous silica or carbons [6,7].

Metal-organic frameworks (MOFs) are considered as an intriguing and a relatively new class of highly-crystalline porous solids that have been gaining a great deal of research interest over the last decade [8,9]. MOFs are versatile materials with high porosity, tunable pore size/shape, and various functional groups, and can be readily tailored for specific applications, such as gas storage, adsorption, and catalysis [10,11,12]. Recently, MOFs are also used as precursors or templates for preparation of nano-metal oxides or nanoporous carbon [13,14]. During thermal treatment in the presence of air, the organic linkers (existing in MOFs) are converted to CO_2_, leaving metal oxides behind [15]. On the other hand, thermal treatment of MOFs under N_2_ atmosphere leads to the formation of metal oxide-carbon composite materials as the organic linkers are transformed into carbon phases with highly-porous structural properties [16].

The rising levels of water pollution are posing a significant health threat with rapid urbanization, industrialization, and population growth [11]. In particular, contamination of water resources by dyes, pharmaceutical, and personal care products (PPCPs), has become a great concern due to their massive use in textile, leather, dyeing, food processing, and pharmaceutical or cosmetic industries [17,18]. Dyes in water are easily recognizable, even in very small quantities, due to their high colorant visibility. Some dyes are toxic, carcinogenic, and interfere with aquatic photosynthesis [17]. Crystal violet (CV, structure is given in Figure S1 in Supplementary Material) is a commonly used dye for coloring cotton and silk. CV is persistent, non-biodegradable, carcinogenic, and can cause skin irritation, respiratory or kidney failures when overexposed [19].

The occurrence of PPCPs has been detected in surface and groundwater since the 1960s, and recently, some of these bio-accumulated PPCPs have been found in the tissues of fish [20]. Salicylic acid (Figure S1) is one of the most widely used PPCPs, and has been frequently detected in natural water resources [21]. SA is mainly used as an intermediate for organic synthesis, to prepare cosmetics and medicines [22,23]. It can cause headache and nausea, and interferes with the normal functions of liver and kidney of the human being [23].

A number of methods have been applied to address dyes and PPCPs contamination, including coagulation [24], photo-degradation [25], advanced oxidation processes, and ozonation [26,27]. Chemical oxidation assisted by UV and ozonation has been successfully applied for removal of dyes and PPCPs, but they are energy and cost intensive, in many cases, and can produce residual toxic byproducts [26,27]. Adsorption, despite its problems related to disposal and post-contamination by spent adsorbents, is considered to be an effective and simple method requiring less operation and energy costs, and many recent investigations have focused on the development of efficient adsorbents for target contaminants.

In this study, Zr-based MOF, UiO-66 (UiO stands for University of Oslo: contains hexa-nuclear zirconium clusters linked by terephthalates [28]) was prepared and subsequently calcined under N_2_ atmosphere to prepare ZrO_2_-carbon (ZC) composites. N-functionalized ZC composite was also prepared from amino-functionalized UiO-66 (NH_2_-UiO-66) precursor under the same condition. The composites were characterized through various instrumental analyses, and a series of experiments, including adsorption kinetics, isotherms, and the effect of pH, were performed with CV and SA to demonstrate the potential utility of the composites, and to gain some insights into the mechanisms governing the adsorption processes.

## 2. Results and Discussions

### 2.1. Characterization of the MOFs and ZC Composites

**Figure 1:**
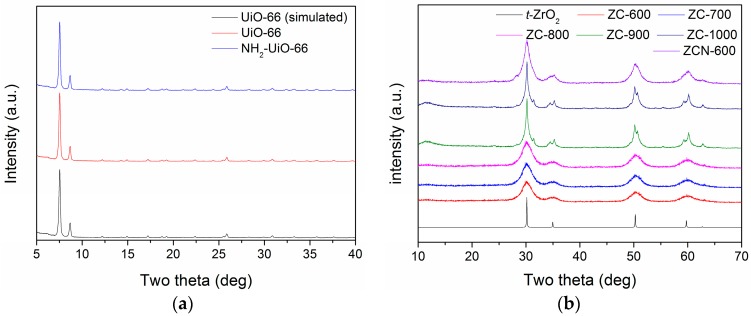
XRD patterns: (**a**) the simulated, virgin, and functionalized UiO-66s; and (**b**) simulated ZrO_2_ and ZC composites.

The results of X-ray powder diffraction (XRD) analysis of the MOFs are presented in Figure 1a. The XRD patterns of both UiO-66 and NH_2_-UiO-66 matched well with that of simulated UiO-66, suggesting there was no significant change in the crystal structure of UiO-66 upon functionalization with NH_2_ group. The FTIR spectra of the NH_2_-funcitionalized MOFs showed C–N stretching and N–H wagging bands at 1258 and 764 cm^−1^, respectively, which signifies the successful incorporation of the NH_2_ in UiO-66 (Figure S2) [29]. Figure 1b shows the XRD patterns of the ZC composites prepared by calcination of UiO-66 at different temperatures under N_2_ atmosphere. The structure of UiO-66 was completely collapsed during calcination at 600 °C and new diffraction peaks appeared at 2θ = 30.4°, 35.3°, 50.7°, and 60.4°, which, respectively, corresponds to the (111), (200), (220), and (311) lattice planes of tetragonal ZrO_2_ (*t*-ZrO_2_) [30]. These characteristic peaks of *t*-ZrO_2_ increased with the increase of the calcination temperature. The carbon phase in the composites appeared to be mostly amorphous up to 800 °C as there were no peaks corresponding to the crystalline carbon phase. Meanwhile, at a calcination temperature ≥900 °C, a peak is observed at 11.2°, which is similar to the diffraction position of the graphite oxide (GO) phase [31]. However, the peak was not very sharp and, thus, there may exist a small amount of amorphous GO layer or, more likely, to have amorphous carbon containing some oxygen groups.

**Figure 2:**
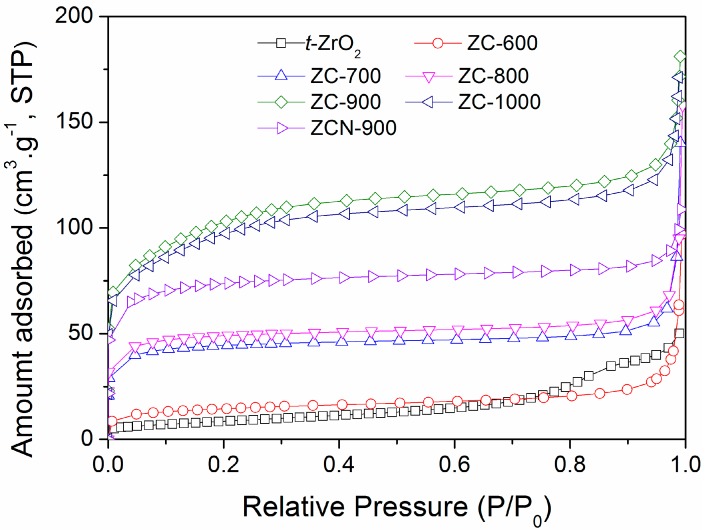
Nitrogen adsorption isotherms of *t*-ZrO_2_ and the ZC composites.

Figure 2 shows the nitrogen adsorption isotherms of the ZC composites, along with ZrO_2_, and their textural properties are summarized in Table 1. With the increase of temperature, the Brunauer-Emmett-Teller (BET) surface area increased significantly; however, the shapes of the nitrogen adsorption envelopes remained unchanged. The maximum surface area was found for ZC-900 (370 m^2^·g^−1^). The surface area ZCN-900 was smaller than that of ZC-900, and this may be due to the presence of N moieties on the composites that physically blocked the surface and pores.

**Figure 3:**
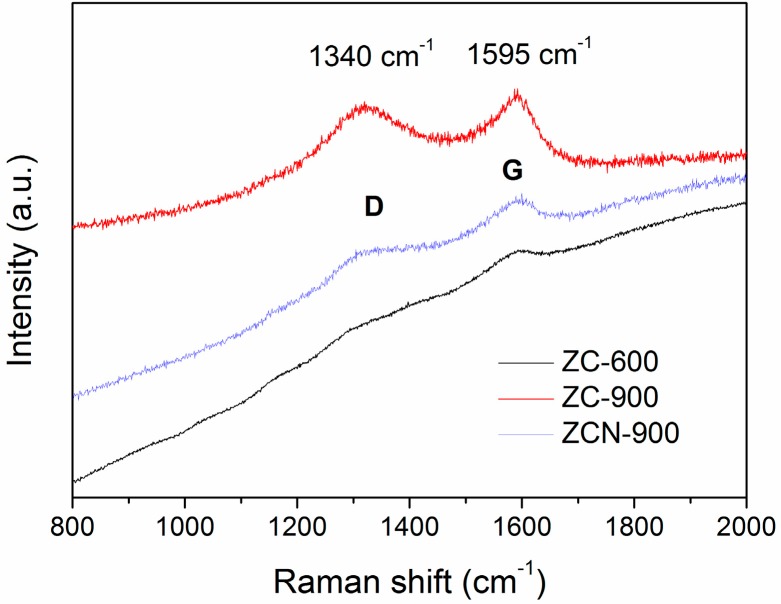
Raman spectra of ZC-900, ZC-900, and ZCN-900.

**Figure 4:**
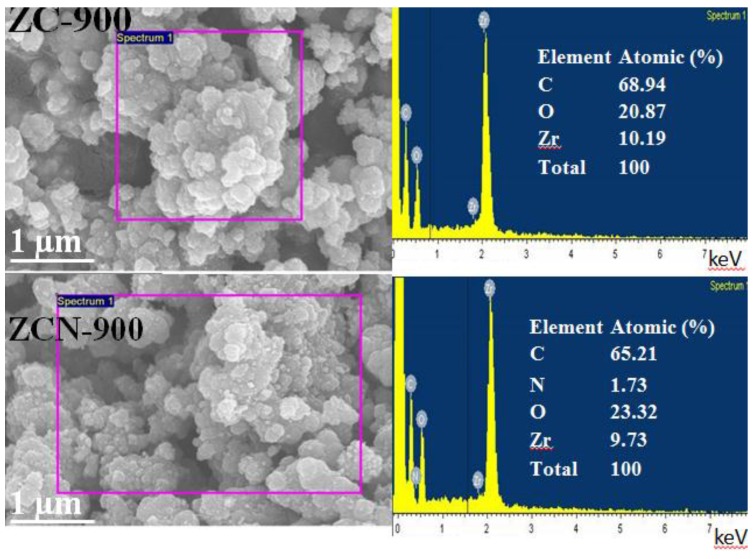
Images of ZC-900 and ZCN-900 obtained using field emission scanning electron microscope coupled with energy-dispersive X-ray spectroscopy (FE-SEM/EDS).

The Raman spectra of ZC-600, ZC-900, and ZCN-900 are presented in Figure 3. All of the spectra possess peaks at 1595 cm^−1^ (known as G bands), which is a characteristic peak for sp^2^ hybridized C–C bonds [32], and the intensity of G bands were observed to increase with the increase in temperature. However, except ZC-600, the other samples showed an additional peak around 1342 cm^−1^ (known as D bands), which related to the defects and disorder in the planar carbon network [32,33]. Figure S3 shows the Field emission scanning electron microscope (FE-SEM) images of ZC-600 and ZC-800, both of which possess nano-sized particles of 80–120 nm with irregular morphology and varying degrees of agglomeration. The agglomeration became more apparent with increasing temperature. The EDS spectrums showed the presence of Zr in ZC-900 and ZCN-900 composites, and N (around 1.7%) in ZCN-900, which demonstrated N-associated functionalization on the ZC surface (Figure 4). The Zr/N ratio for ZCN-900 was 5.62 before the adsorption. This ratio remained relatively constant (5.52) after adsorption of CV (Figure S4).

### 2.2. CV Adsorption on the Composites

**Figure 5:**
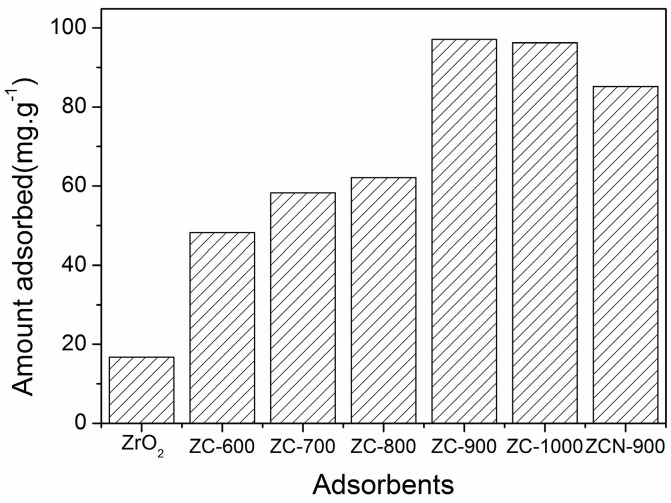
Adsorption of CV on different adsorbents. Initial CV concentration = 25.1 mg·L^−1^, 24 h reaction time.

The results of CV adsorption on ZrO_2_ and the ZC composites are presented in Figure 5. The adsorption of CV by the composites was much higher than that by ZrO_2_, and it increased with an increase of calcination temperature of the composites up to 900 °C. A further increase of calcination temperature to 1000 °C did not have significant effects on the textural properties or adsorption capacity (Table 1 and Figure 5). In addition, the functionalized ZCN-900 exhibited a slightly lower adsorption capacity compared to ZC-900.

Calcination temperature has been reported to have a significant effect on the surface area and pore volume, as well as the phase selectivity, of carbonaceous materials [34]. A similar effect of increasing temperature was also observed for the ZC composites, leading to the increase of the surface area and pore volume (Table 1). In a similar context, the reduced CV adsorption on functionalized ZCN-900, as compared to ZC-900, could be attributed to the reduction in surface area and pore volume as a result of N-functionalization. In addition, a new phase of GO was evolved during calcination at 900 °C, which presumably contributed to the adsorption by ZC-900. It has been reported that the presence of GO on the adsorbent enhances the adsorption of cationic dye since it imparts surface acidity and functional groups that could lead to additional specific interactions with ionic contaminants [35].

**Figure 6:**
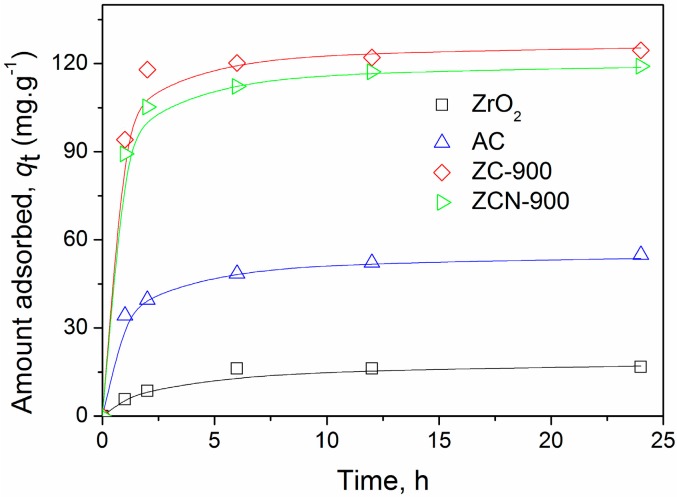
Effect of contact time on adsorption of CV on different adsorbents (initial CV concentration = 50.2 mg·L^−1^). The solid lines show the calculated results derived from pseudo-second order non-linear method.

CV adsorption of ZrO_2_, ZC-900, and ZCN-900 was further evaluated in kinetics experiments, along with a commercial activated carbon (AC) for comparison purposes (Figure 6). Rapid adsorption occurred in the initial phase for all of the adsorbents, but site saturation started to occur at later times before reaching equilibrium. The adsorption of CV on ZC-900 and ZCN-900 were significantly greater than that of AC, despite their smaller surface area and total pore volume (Table 1). This suggests that specific interactions involving surface functional groups played an important role in overall adsorption of CV on the ZC composites. For ZC-900 and ZCN-900, adsorption of CV was close to completion after 2 h whereas, for AC, adsorption steadily proceeded until 12 h. A pseudo-second-order non-linear kinetic model [29] was applied to the kinetics data in Figure 6, and the rate constants (*k*_2_) are listed in Table 1. The high correlation factors (*r*^2^) indicate the kinetics data are in good agreement with the model.

### 2.3. Adsorption Isotherms

**Figure 7:**
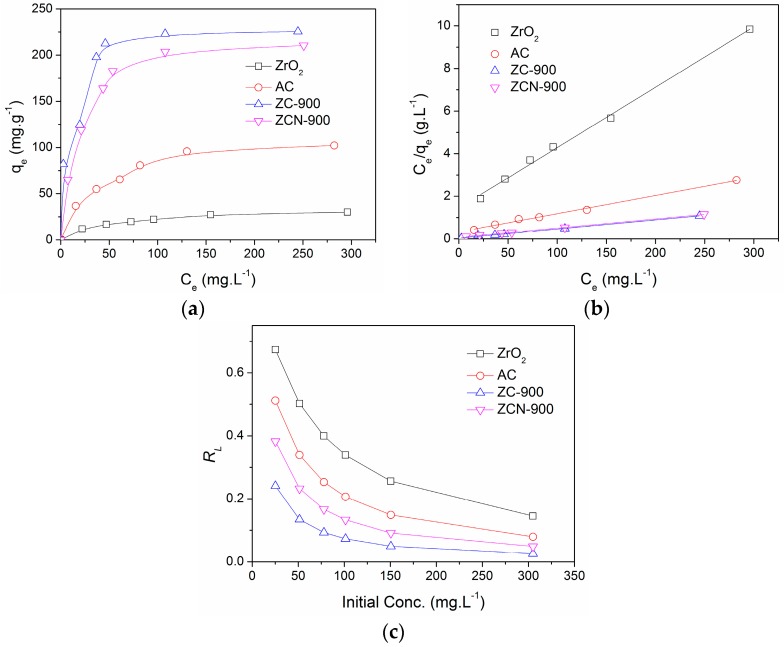
(**a**) Adsorption isotherms; (**b**) Langmuir plots; and (**c**) effects of initial concentrations on *R_L_* for the adsorption of CV on different adsorbents.

The results of isotherm experiments in the CV concentration range of 25–300 mg·L^−1^ are presented in Figure 7a. The ZC-900 and ZCN-900 showed greater adsorption capabilities than ZrO_2_ and AC. Langmuir plots (Figure 7b) were used to determine the maximum adsorption capacity (*Q*_0_), [29], and the values are presented in Table 1. ZC-900 (243 mg·g^−1^) showed a slightly higher adsorption capacity compared to ZCN-900 (222 mg·g^−1^). Table 2 also summarizes *Q*_0_ values of various adsorbents used for CV adsorption in the literature, and it indicates ZC-900 has a competitive adsorption capability to other adsorbents. Figure 7c shows the calculated *R*_L_ values [36] as a function of the initial CV concentrations for four different adsorbents. *R*_L_ values fell within the range of 0–1, indicating a favorable adsorption of CV for all four adsorbents [36]. In particular, *R*_L_ values of ZC-900 were smallest in all concentration ranges, and this means CV adsorption occurred most readily on ZC-900.

**Figure 8:**
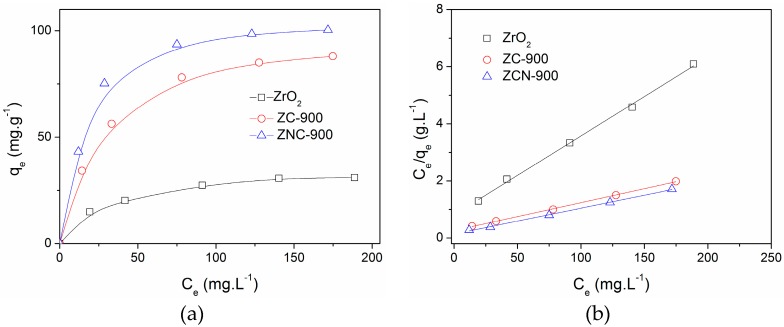
(**a**) Adsorption isotherms; and (**b**) Langmuir plots for the adsorption of SA on different adsorbents.

The results presented above demonstrated the ZC composites are very efficient in removing a cationic dye. To further probe the capability of the composites, the composites were applied to adsorption of SA, one of the most commonly found PPCPs in wastewaters. The adsorption isotherms for three adsorbents are plotted in Figure 8a, along with Langmuir model fittings (Figure 8b), and *Q*_0_ values calculated from the model are presented in Table 3. The *Q*_0_ value of SA adsorption on ZC-900 was approximately three times higher than that on ZrO_2_, similar to the case of CV adsorption. In addition, despite its smaller surface area and pore volume, ZCN-900 showed a better performance than ZC-900, signifying the contributing role of N-functionalization in the overall absorption of SA.

### 2.4. Adsorption Mechanisms

**Figure 9:**
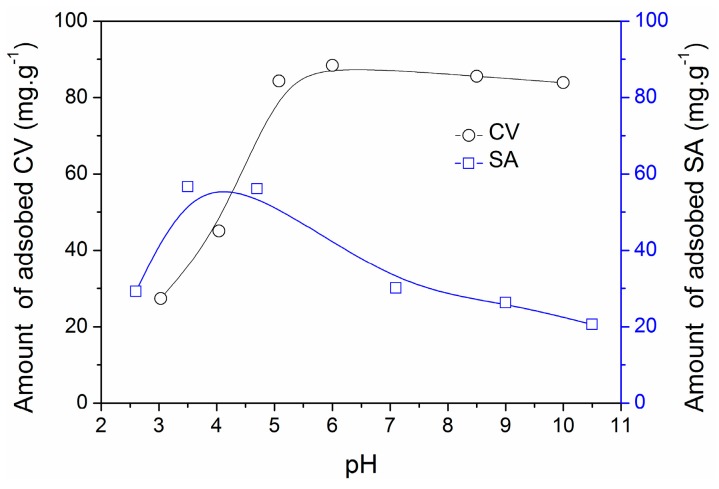
Effect of pH on adsorption of CV (25.1 mg·L^−1^) and SA (50.2 mg·L^−1^).

Several possible interactions have been suggested to explain adsorption of organic contaminants on carbonaceous materials, including non-specific hydrophobic bonding, electrostatic interactions, hydrogen bonding, and π–π staking [38,42]. In addition, adsorption of ionic contaminants on ZrO_2_ has been reported to occur via electrostatic interactions [4]. The molecular size of CV (11.13 ‎Å) and SA (5.10 ‎Å) is much smaller than the mean pore size of the composites (Table 1), which suggests adsorption through pore diffusion could occur. Figure 9 shows the effect of pH on the adsorption of CV and SA by ZC-900. The pH at the point of zero-charge (pH_pzc_) of ZC-900 was found to be ~4.9 (Figure S5). As a consequence, the surface of ZC-900 becomes positively charged at solution pH < 4.9, and the charge density increases with the decrease of solution pH. The effect of solution pH on CV adsorption was clearly observed from the Figure 9 where the adsorbed quantity decreased sharply from 84.3 to 27.3 mg·g^−1^ with the pH decrease from 5 to 3. This might be attributable to the increase of positive charge density on the adsorbent surface that partially obstructed the entrance of cationic CV molecules to the binding sites. Meanwhile, CV adsorption showed little pH dependence at pH 6 to 10 despite the increasing negative charge density on the surface, suggesting an electrostatic interaction was not the dominant mechanism for CV adsorption. This means ZrO_2_ incorporated in ZC-900 had a limited contribution to overall adsorption of CV, but rather its adsorption is more likely to be controlled by interactions associated with carbon in the composite. In addition, CV is a large molecule that consists of triphenyl backbones with three bulky dimethylamino groups. This bulky structural property may exert a steric hindrance to electrostatic bonding to functional groups on ZrO_2_ or carbon surface.

On the other hand, SA adsorption showed a very responsive variation to pH change. It increased from 29.2 to 56 mg·g^−1^ with the increase of pH 2.6 to 4.7, respectively, but gradually decreased at pH > 4.7 to yield the lowest adsorption of 20.6 mg·g^−1^ at pH 10.5. The *pk_a_* value of SA is 2.9, which means SA preferably exists in ionic form at pH > 2.9. Therefore, the favorable electrostatic interactions may exist between SA anions and the positively charged surface of ZC-900 (up to pH~4.9). However, both SA and ZC-900 become negatively charged at pH > 4.9, which would result in a reduction of its adsorption. Moreover, the decreased adsorption at pH < 2.5 could be explained by the speciation of the molecule to neutral or un-ionized forms at such pH conditions. This pH dependence indicates that SA adsorption is closely related to the surface charge of adsorbent. In other words, contrary to the adsorption mechanism of CV, electrostatic attraction exerted by ZrO_2_ and various functional groups on the carbon played a key role in the adsorption of SA. Furthermore, it is possible that an ion-exchange reaction between Zr-ligand (-OH) and anionic SA could contribute to the adsorption. Additionally, the presence of base-base repulsion or hydrogen bonding cannot be ruled out during adsorption of CV or SA on ZCN-900. The reduced adsorption of CV on ZCN-900 may result from the existence of base-base repulsion between the N moieties of CV and those of ZCN-900 (Figure S1). On the other hand, a little enhancement in the adsorption of SA on ZCN-900 could be explained by hydrogen bonding between -OH groups of SA and N moieties of ZCN-900.

## 3. Materials and Methods

### 3.1. Materials

All solvents and reagents were purchased from commercial vendors and used without further purification. Zirconium chloride (ZrCl_4_, 99.5%), 1,4-benzenedicarboxylic acid (terephthalic acid, C_8_H_6_O_4_, H_2_BDC, C_8_H_6_O_4_, 98.0%), amino-terephthalic acid (NH_2_-BDC, C_8_H_7_NO_4_, 99.0%), and SA C_7_H_6_O_3_, 99.0%) were purchased from Alfa Aeser, Ward Hill, MA, USA. CV (C_25_N_3_H_30_Cl, ≥90.0%) and ZrO_2_ (99.9%) were bought from Sigma Aldrich, St. Louis, MO, USA. Hydrochloric acid (HCl, 36.0%), nitric acid (HNO_3_, 60.0%), methanol (CH_3_OH, >99.5%), and *N*,*N*-dimethylformamide (DMF, C_3_H_7_NO, 99.0%) were obtained from OCI Chemicals. Activated carbon (AC, granule: size 2–3 mm) was purchased from Duksan Chemical (Ansan, Korea).

### 3.2. Synthesis of UiO-66 and NH_2_-UiO-66

The synthesis of UiO-66 and NH_2_-UiO-66 was conducted according to the procedure by Cavka *et al.* [28], with a minor modification. In brief, 2 mmol ZrCl_4_, 4 mmol H_2_BDC, 2 mmol HCl, and 50 mL DMF were added to a Teflon-lined autoclave (volume: 100 mL), and heated in an electric oven for 24 h at 120 °C. For the synthesis of NH_2_-UiO-66, the same molar amount of NH_2_-BDC was used in place of H_2_BDC. After the synthesis, the resulting solid was filtered and purified with DMF (1 g MOF in 50 mL DMF and heated at 150 °C for 5 h). Finally, the products were filtered and dried in the oven at 150 °C for 12 h.

### 3.3. Preparation of ZC Composites

The synthesized MOFs (UiO-66 and NH_2_-UiO-66) were taken in an alumina crucible, which was placed in a tubular reactor for calcination. A quartz tubing with a dimension of 25.4 mm outer diameter and 610 mm length (Chemglass CGQ-0900T-13) was used as a tubular reactor and assembled using a stainless Ultra Torr Vacuum Fitting (Swagelok SS-4-UT-6-400). The MOFs were heated at a temperature ranging from 600 to 1000 °C for 4 h using a split-hinged furnace (AsOne, Osaka, Japan). The heating rate was 5 °C·min^−1^ and temperature was monitored by an S-type thermocouple. The N_2_ gas flow rate was fixed at 500 mL·min^−1^ using a Brooks mass flow controller (5850 series E, Brooks Instrument, Hatfield, PA, USA ), and a computer-aided control system by LabVIEW (National Instrument, Austin, TX, USA) was employed to control the total procedure. After being cooled to room temperature, a black powder was obtained. The samples obtained from UiO-66 and NH_2_-UiO-66 was denoted as ZC-X and ZCN-X, respectively, where X stands for calcination temperature.

### 3.4. Characterization of Adsorbents

X-ray powder diffraction (XRD) analysis was conducted with the Rigaku DMax-2500 diffractometer (Rigaku, Woodlands, TX, USA) using CuKα radiation. The surface area and pore size of the prepared samples were obtained from the nitrogen adsorption method using a BELSORP-mini II (MicrotracBEL, Osaka, Japan). The samples were evacuated at 150 °C for 12 h before nitrogen adsorption at −196 °C. The morphologies and composition of the samples were examined with a field emission scanning electron microscope (FE-SEM, JEOL-JSM7401F, Peabody, MA, USA ), coupled with energy-dispersive X-ray spectroscopy (EDS, JEOL). The Raman spectrum was taken using a inVia reflex Raman microscope (Renishaw, Gloucestershire, UK). The Fourier transform infrared spectroscopy (FTIR) was measured using a Spectrum 100 spectrometer (PerkinElmer, Waltham, MA, USA). The molecular sizes of CV and SA were calculated using Chem3D software (CambridgeSoft, Waltham, MA, USA).

### 3.5. Zero Point Charge pH Measurement

The pH at the zero point charge of ZC-900 was measured by means of the pH drift method according to the reported literature with slight modification [43,44,45]. 10 mL of 0.01 M NaCl solution was taken in a 20 mL vial and pH was adjusted to a value between 2 and 12 by using HCl 0.1 M or NaOH 0.1 M solution. Then, 0.03 g of ZC-900 was added in each vial and shaken for 48 h at room temperature. The final solution pH was measured and was plotted against the initial pH. The point where the plot crossed the initial pH = final pH line was considered as the pH at the zero point charge and it was found to be 4.9 for the ZC-900.

### 3.6. Adsorption Experiments

Stock solutions of CV (1000 mg·L^−1^) and SA (500 mg·L^−1^) were prepared by dissolving 1 g of CV and 0.5 g SA in 1 L deionized distilled water, respectively. Prior to adsorption, the adsorbents were dried for 12 h at 100 °C. All of the adsorption experiments, including kinetics, isotherms, and the effect of pH, were conducted in batch reactors using 5 mg adsorbents in 20 mL solution. Kinetics experiments were carried out with 50.2 mg·L^−1^ CV solutions, and the reactors were agitated at 200 rpm at room temperature (23 ± 2 °C). The supernatants were collected at predetermined intervals and filtered with 0.45 μm filter (25 mm GD/X disposable filter, polyvinylidene difluoride membrane, 0.45 μm, (Whatman, Pittsburgh, PA, USA). Adsorption isotherm experiments were conducted in concentration ranges of 25–300 mg·L^−1^ for CV and 25–200 mg·L^−1^ for SA. The concentrations of CV and SA were measured using a UV–VIS spectrophotometer (Hach DR/4000, Loveland, CO, USA) at wavelengths of 590 and 290 nm, respectively. To determine the amount of adsorbed CV or SA, the following mass-balance relation (Equation (1)) was used:

(1) $$\frac{C_{e}}{q_{e}}=\frac{C_{e}}{Q_{0}}+\frac{1}{Q_{0}b}$$

where *C*_0_ (mg·L^−1^) is the initial concentration, *C_t_* (mg·L^−1^) is the concentration at time *t*, *V* (L) is the volume of the CV solution and *w* (g) is the weight of the adsorbents. The solution pH was measured using a pH meter (Horiba, Ltd., Kyoto, Japan). The effect of pH was also investigated in pH ranges of 2–10 using 25.1 mg·L^−1^ CV and 50.2 mg·L^−1^ SA after 24 h reactions. The initial pH of the solutions were adjusted by using 0.1 N HCl or 0.1 N NaOH. All of the experiments were conducted in duplicate, and the relative errors were less than 5%. The experimental data were analyzed with a pseudo-second-order non-linear kinetic model, Langmuir isotherms, and separation factors (*R*_L_). Details of these methods are provided in Supplementary Materials.

### 3.7. Calculation of Adsorption Kinetics

The adsorption kinetics were interpreted with a pseudo-second-order non-linear kinetic model [46,47] which is expressed by the following equation:

(2) $$q_{t}=\frac{q_{e}^{2}k_{2}t}{1+q_{e}k_{2}t}$$

where, *q_e_* (mg·g^−1^): amount adsorbed at equilibrium; *q_t_* (mg·g^−1^): amount adsorbed at time *t*; *t* (h): adsorption time; and *k*_2_ (g mg^−1^·h^−1^): the pseudo-second-order rate constant.

A trial-and-error procedure was adapted to measure the pseudo-second order kinetic parameters in the case of the non-linear method using the solver add-in with Microsoft's spreadsheet program, Microsoft Excel.

### 3.8. Calculation of Maximum Adsorption Capacity (Q_0_) and Separation Factor (R_L_)

The maximum adsorption capacity was calculated using the Langmuir adsorption isotherm [48,49] after adsorption for 24 h. The adsorption isotherms under various conditions were plotted to follow the Langmuir equation [48,49]:

(3) $$\frac{C_{e}}{q_{e}}=\frac{C_{e}}{Q_{0}}+\frac{1}{Q_{0}b}$$

where, *C_e_*: equilibrium concentration of adsorbate (mg·L^−1^); *q_e_*: amount adsorbed at equilibrium (mg·g^−1^); *Q*_0_: maximum adsorption capacity (mg·g^−1^); and *b*: Langmuir constant (L·mg^−1^).

Therefore, the maximum adsorption capacity *Q*_0_ can be obtained from the reciprocal of the slope of the plot of *C_e_*/*q_e_* against *C_e_*. The *b* value was obtained from the intercept (1/*Q*_0_*b*) of the Langmuir plot.

Separation factor (*R*_L_) [50,51,52] was calculated using the following equation to understand further the adsorption process:

$$R_{L}=\frac{1}{1+bC_{0}}$$

where, *R*_L_: separation factor; *b*: Langmuir constant (L·mg^−1^); and *C*_0_: initial concentration of adsorbate (mg·L^−1^)

## 4. Conclusions

ZC- and N-functionalized ZC composites were prepared successfully using UiO-66 and NH_2_-UiO-66 via calcination under N_2_ atmosphere. It was found that 900 °C was the optimal temperature to form high-surface-area ZC composites possessing a small amount of amorphous GO or some oxygen containing carbon phase. CV adsorption followed the pseudo-second-order kinetic model and the *k*_2_ value increased in the order of ZrO_2_ < AC < ZCN-900 < ZC-900. The ZC-900 composite also showed maximum uptake of 243 mg·g^−1^ CV, which is approximately 7 and two times higher than the adsorption by ZrO_2_ and AC, respectively. The higher adsorption of CV on ZC-900 could be attributed to the synergic effect of small-sized and well-dispersed ZrO_2_ over the highly porous surface. The prepared composites also showed a good performance for adsorption of SA. The favorable adsorption of CV was observed at pH > 4.9, whereas optimal removal of SA was achieved in the pH range of 3.1–4.7. Specific interactions involving functional groups on the ZC composites appeared to control the CV adsorption, while electrostatic interactions are assumed to play a key role in the adsorption of SA. Furthermore, in the case of ZCN-900, base-base repulsion and hydrogen bonding are likely to influence the adsorption of CV and SA, respectively, to some extent.

## Figures and Tables

**Table 1:** Textural properties and the results for the CV adsorption on different adsorbents.

Adsorbent	BET Surface Area (m^2^·g^−1^)	Total Pore Volume (cm^3^·g^−1^)	Mean Pore Size (nm)	Pseudo-Second-Order Kinetics Parameters ^a^ (g·mg^−1^·h^−1^)		Q_0_^b^ (mg·g^−1^)	b ^c^ (L·mg^−1^)	*r*^2^ (Langmuir Plot)
				*k*_2_	*r*^2^			
AC	870	0.44	3.6	0.016	0.999	116	0.019	0.995
ZrO_2_	30	0.44	-	0.022	0.989	35	0.039	0.996
ZC-600	56	0.11	1.5	-	-	-	-	-
ZC-700	168	0.15	1.7	-	-	-	-	-
ZC-800	184	0.16	1.9	-	-	-	-	-
ZC-900	370	0.26	2.9	0.027	0.998	243	0.119	0.996
ZC-1000	345	0.25	2.2	-	-	-	-	-
ZCN-900	276	0.17	2.3	0.025	0.999	222	0.064	0.998

^a^ Derived from non-linear model using a Microsoft Excel add-in solver; ^b^ Maximum adsorption capacity; ^c^ Langmuir constant.

**Table 2:** A comparison of maximum adsorption capacity of ZC composites with other adsorbents for the aqueous phase adsorption of CV.

Entry	Adsorbents	Maximum Adsorption Capacity (mg·g^−1^)	Reference
1	Cellulose-based adsorbent	182	[37]
2	Magnetic nanocomposite	113	[38]
3	Chitosan-graphite oxide modified polyurethane foam	58	[39]
4	Mycelial biomass	239	[40]
5	Palm kernel fier	79	[41]
6	Activated carbon (commercial)	116	This study
7	ZC-900	243	This study

**Table 3:** Maximum adsorption capacities and Langmuir parameters for the adsorption of SA on different adsorbents.

Adsorbents	*Q*_0_^a^ (mg·g^−1^)	*b* ^b^ (L·mg^−1^)	*r*^2^ (Langmuir Plot)
ZrO_2_	36	0.034	0.998
ZC-900	102	0.371	0.996
ZCN-900	109	0.047	0.998

^a^ Calculated from Langmuir plots; ^b^ Langmuir constant.

## Supplementary Materials

The following are available online at www.mdpi.com/1996-1944/9/4/261/s1. Figure S1: Structures of crystal violet (CV) and salicylic acid (SA), and plausible interactions between N-functionalized zirconia-carbon composites ZCN composites and adsorbates.; Figure S2: Fourier transform infrared spectroscopy (FTIR) spectra of UiO-66 and NH2-UiO-66 (UiO stands for University of Oslo).; Figure S3: Field emission scanning electron microscope (FE-SEM) images of ZC-600, ZC-800, ZC-900, and ZNC-900.; Figure S4: Images of ZC-900 after adsorption of CV obtained from using field emission scanning electron microscope coupled with energy-dispersive X-ray spectroscopy (FE-SEM/EDS); Figure S5: Determination of the pH at the zero point charge for ZC-900 by the pH drift method.
