# Supplementary material for: Preparation of Calcined Zirconia-Carbon Composite from Metal Organic Frameworks and Its Application to Adsorption of Crystal Violet and Salicylic Acid
Source: Materials (Basel). 2016 Mar 31;9(4):261. doi: 10.3390/ma9040261 (PMC5502925; doi:10.3390/ma9040261)
Supplement: Supplementary file 1 [file materials-09-00261-s001.pdf]

Supplementary Materials: Preparation of Calcined Zirconia-Carbon Composite from Metal Organic Frameworks and Its Application to Adsorption of Crystal Violet and Salicylic Acid

Zubair Hasan, Dong-Wan Cho, In-Hyun Nam, Chul-Min Chon and Hocheol Song

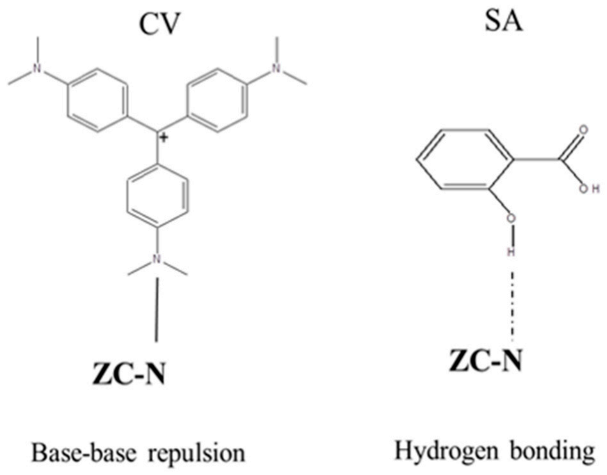

Figure S1. Structures of crystal violet (CV) and salicylic acid (SA), and plausible interactions between N-functionalized zirconia-carbon composites ZCN composites and adsorbates.

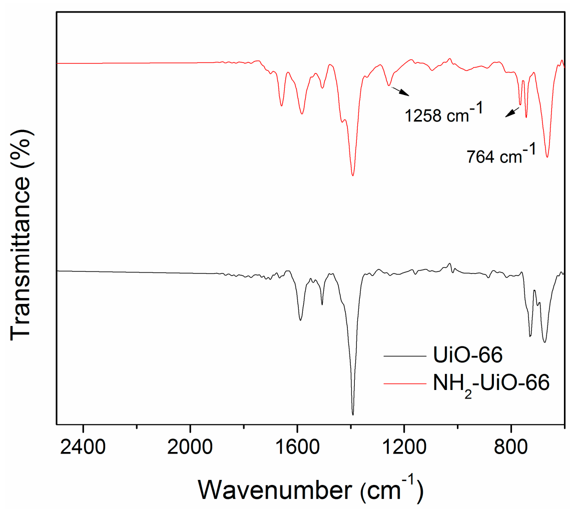

Figure S2. Fourier transform infrared spectroscopy (FTIR)spectra of UiO-66 and NH_2-UiO-66 (UiO stands for University of Oslo).

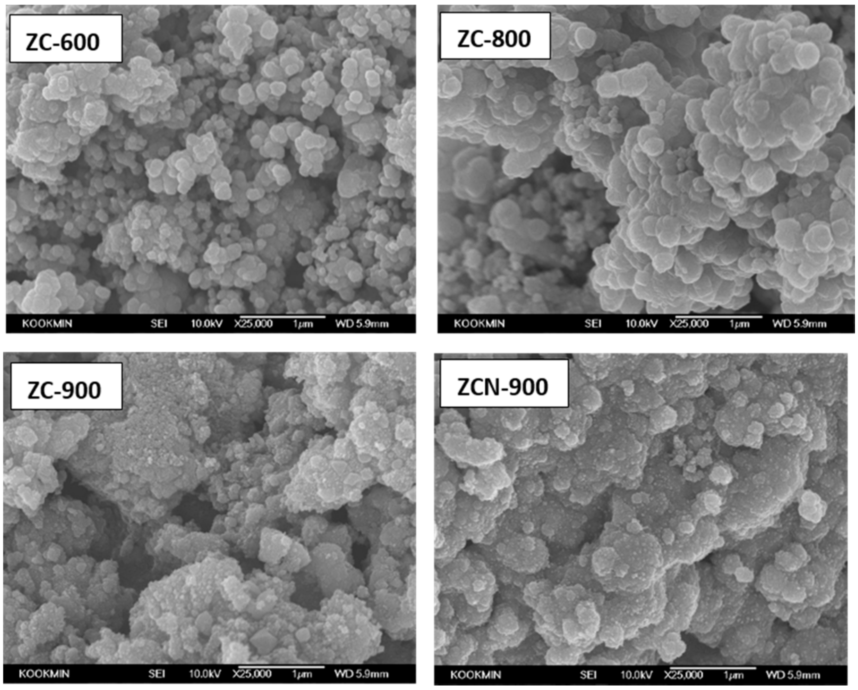

Figure S3. Field emission scanning electron microscope (FE-SEM) images of ZC-600, ZC-800, ZC-900, and ZNC-900.

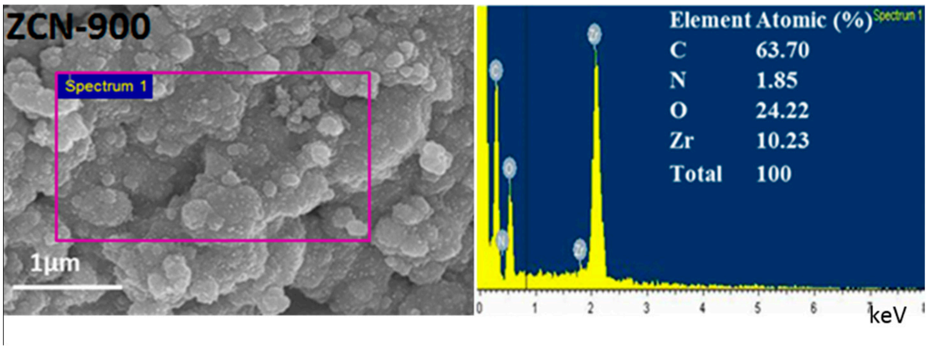

Figure S4. Images of ZC-900 after adsorption of CV obtained using field emission scanning electron microscope coupled with energy-dispersive X-ray spectroscopy (FE-SEM/EDS).

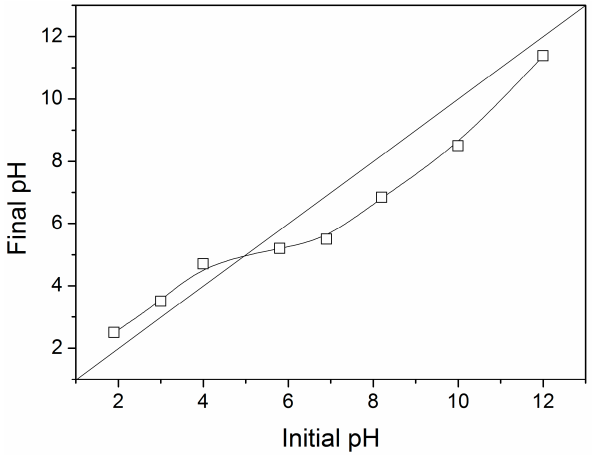

Figure S5. Determination of the pH at the zero point charge for ZC-900 by the pH drift method.
